# Anatomic feasibility of the WeFlow-JAAA endograft system for treating juxtarenal and pararenal abdominal aortic aneurysms

**DOI:** 10.1038/s41598-025-23485-y

**Published:** 2025-11-13

**Authors:** Jiang-Ping Gao, Chun-An-Sheng Wang, Hong-Peng Zhang, Li-Jun Wang, Wei Guo

**Affiliations:** 1 Department of Vascular Surgery, Chinese PLA General Hospital, No.28 Fu-xing Road, Beijing, 100853 China; 2 Medical School of the Chinese PLA, Beijing, China

**Keywords:** Aortic aneurysm, Abdominal, Anatomy, Juxtarenal, Pararenal, Off-the-shelf, Aneurysm, Aortic diseases

## Abstract

This study aimed to evaluate the anatomic feasibility of a novel off-the-shelf device (modular endograft with superior mesenteric artery fenestration, celiac scallop, and bilateral renal inner branches) designed for the treatment of juxtarenal and pararenal abdominal aortic aneurysms (AAAs). Digital computed tomography angiograms were analyzed from 170 consecutive patients with juxtarenal or pararenal AAAs who participated in imaging screening for a national multicenter clinical trial assessing the effectiveness of the WeFlow-JAAA system between February 2022 and January 2024. Anatomic feasibility was evaluated in accordance with the investigational protocols and the instructions for use. A proximal seal of ≥ 20 mm was achieved in 98.2% of patients, and renovisceral artery incorporation was observed in 73.5% (125/170) of cases. According to the conservative instructions for use (IFU) criteria, anatomic feasibility was achieved in 34.7% (95%CI: 27.5%~41.9%) of patients. When applying the liberal IFU criteria, the suitability rate increased to 64.1% (95%CI: 56.8%~71.4%), with configurations A and B being suitable for 56.5% and 60.6% of cases, respectively. The WeFlow-JAAA endograft system exhibits a suitability rate of 64.1% for treating JRAAAs and PRAAAs with a healthy sealing zone of at least 5 mm below the SMA within the Chinese population. Nevertheless, the current design does not optimally allow for the incorporation of renovisceral arteries, indicating a need for further technical research in this domain.

**Supplementary Information:**

The online version contains supplementary material available at 10.1038/s41598-025-23485-y.

## Introduction

 Anatomical constraints, particularly the presence of a suboptimal proximal neck morphology, significantly impede the feasibility of endovascular aortic repair (EVAR) in nearly half of patients with abdominal aortic aneurysms (AAAs)^1,2^. Specifically, suboptimal proximal neck morphology is responsible for 50% to 60% of these cases^3^. In response to these challenges, fenestration techniques, especially customized fenestration, have emerged as a recommended alternative to open repair for AAAs with inadequate proximal necks^4,5^. However, the complexity and specificity of customized fenestration often necessitate the use of company-manufactured devices (CMDs)^6,7^, which typically require a production lead time of three to four weeks. This delay can impede timely treatment for symptomatic complex AAAs and potentially increase the risk of rupture^8^.

To address this limitation, the development of off-the-shelf (OTS) devices that are suitable for the majority of AAA patients with suboptimal proximal necks is essential. For example, the Cook p-Branch (Cook Medical, Bloomington, IN, USA) is an investigational OTS device designed for the treatment of both juxtarenal and pararenal AAAs^9^. Initial studies have demonstrated its technical success and overall feasibility^9–11^. Nevertheless, these devices are currently restricted to investigational use. Furthermore, due to the limited availability of officially sanctioned CMDs in China, many cases of juxtarenal or pararenal AAAs are managed with physician-modified fenestrated stent-grafts, which may pose legal and ethical challenges.

The WeFlow-JAAA stent graft system, an innovative OTS device developed by Endonom Medtech (Hangzhou, China), aims to treat juxtarenal and partial pararenal AAAs. Initial studies have shown promising technical and clinical success^12^, and a large, multicenter, prospective clinical trial (NCT05179967) is nearing completion in China^13^. However, the overall anatomical feasibility of this system has yet to be clarified. The objective of this study was to evaluate the anatomical feasibility of this novel OTS device in the treatment of patients with juxtarenal and pararenal AAAs.

## Methods

### Study design and patients

This study utilized data from the WeFlow-JAAA national multicenter clinical trial database (NCT05179967). The preliminary clinical outcomes of this multicenter clinical trial have just been published online^14^. This study exclusively focuses on anatomical suitability and does not assess or suggest any clinical outcomes, including safety or effectiveness. All patients with juxtarenal abdominal aortic aneurysms (JRAAAs) and pararenal abdominal aortic aneurysms (PRAAAs), who underwent imaging screening prior to enrollment in the multicenter clinical trial between February 2022 and January 2024, were included in the study (Supplementary Fig. S1: Flow diagram). The JRAAA was defined as having a short infrarenal neck (< 10 mm)^4^, while the PRAAA was characterized as an aneurysm extending above one or both renal arteries but not involving the superior mesenteric artery (SMA)^15^. Patients with thoracoabdominal aortic aneurysms or conventional AAAs (proximal neck length ≥ 10 mm) were excluded.

The study received approval (2021-025) from the Institutional Review Board (IRB) of the Chinese PLA General Hospital, which served as the central IRB and obtained acceptance from all participating institutions. All experiments were performed in accordance with relevant guidelines and regulations. Given the minimal-risk nature of this study, the central IRB granted a waiver for informed consent.

### Device design

**Fig. 1:**
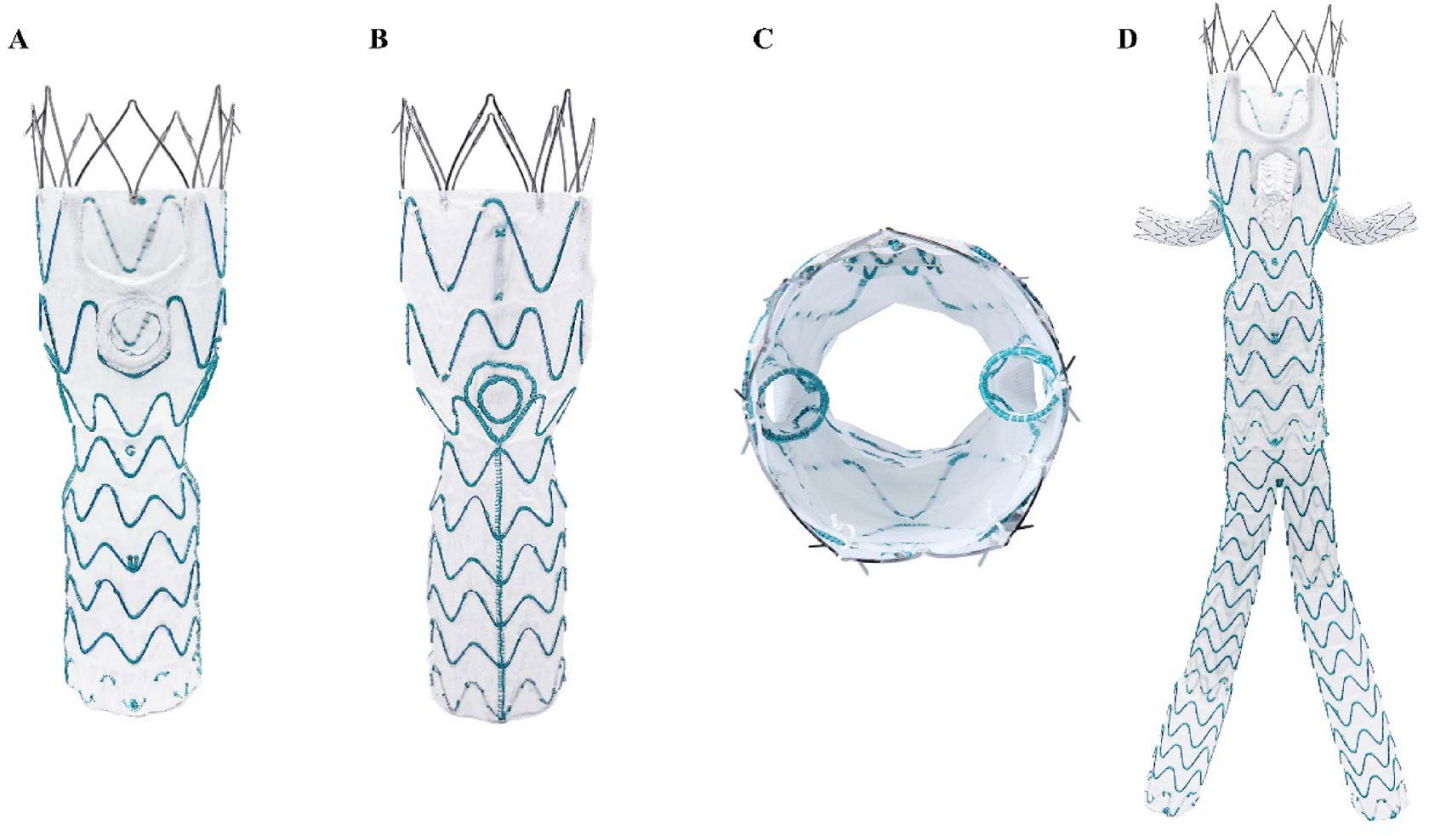
Anterior (**A**), lateral (**B**), and internal (**C**) views of the proximal body of the WeFlow-JAAA off-the-shelf stent system (**D**).

**Fig. 2:**
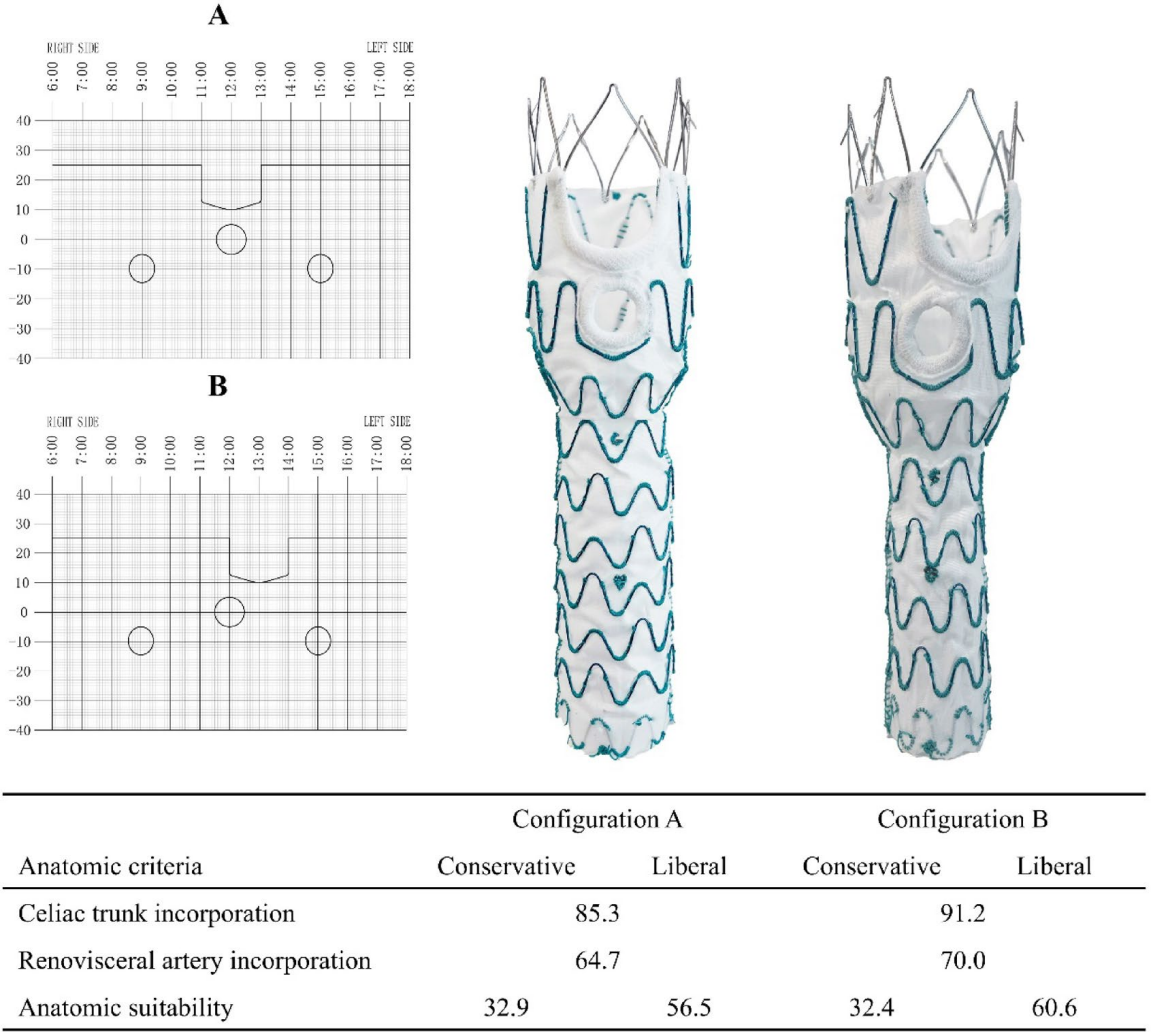
The proximal body sizing sheets, product photos, and the anatomic feasibility of the WeFlow-JAAA for configurations (**A**) and (**B**).

The WeFlow-JAAA system is a modular endograft system composed of four components, and its device specifications and implantation techniques have been previously described in detail^12,13^. Briefly, the proximal component features a mixed design that includes two standard inner branches for both renal arteries, one 3-mm mini-inner-cuff reinforced fenestration for the SMA, and a scallop for the celiac artery (Fig. 1). In design A, the celiac artery scallop is positioned between 11 o’clock and 1 o’clock, while in design B, it is located between 12 o’clock and 2 o’clock (Fig. 2).

### Image analysis and measurements

An experienced investigator (J.P.G.), who was blinded to the enrollment status and clinical variables of all individuals, conducted all image measurements using the 3D Slicer system (version 4.13.0, link: https://www.slicer.org). Two investigators (J.P.G. and C.A.S.W.) then independently analyzed the data. W.G. acted as the adjudicator in the event of disagreement.

All measurements, including the assessment of thrombus, calcium, and aortic circumference, were conducted in accordance with the standardized methods outlined in the Society of Vascular Surgery (SVS) reporting standards^15^. All measurements were based on thin-slice (0.625 mm) CTA data and performed along orthogonal planes reconstructed from the 3D aortic centerline, which was automatically generated using the “*SlicerVMTK*” module in 3D Slicer (v4.13.0). Various measurements were obtained, including the aortic diameter at the level of each target vessel, the length of the effective proximal sealing zone below the SMA, and the longitudinal distances between the lower margin of the celiac trunk and the upper margin of the SMA, as well as between the lower margin of the SMA and the upper margin of both renal artery origins.

The total effective proximal sealing zone was defined as the length of the seal with circumferential fabric apposing the aorta wall^15^, with a standardized length of 20 mm for the WeFlow-JAAA stent-graft system. A healthy proximal sealing zone, or healthy neck, was characterized by a parallel aortic wall with an outer diameter of 18–34 mm, devoid of extensive thrombus (thickness >5 mm in more than 50% of the aortic circumference) and calcium (also >50% of the aortic circumference). The clock positions of the celiac trunk and both renal arteries were determined with the SMA serving as the 12:00 reference point.

Additional anatomic factors measured included the α-angle, γ-angle, non-bifurcated segment length of the target vessels, the take-off angle of renal arteries^15^, aberrant or accessory anatomy, and the diameters of the target vessel and access vessels. The γ-angle was defined as the maximum tortuosity or angulation along the centerline of the proximal landing zone, spanning from 33 to 35 mm proximally to the superior margin of the SMA to 5 mm below the lower margin of the SMA (Supplementary Fig. S2). This measurement encompassed both the proximal sealing zone and the bare stent segment, playing a crucial role in ensuring optimal stent adherence to the aortic wall following the procedure. The α-angle was defined as the maximum angle between the proximal sealing zone and the aortic segment harbouring the renal artery immediately below the proximal sealing zone, which is significant for the successful implantation as well as the long-term patency of the renal artery branches.

### Anatomic criteria

Table 1 presents the anatomic criteria for the WeFlow-JAAA system. In addition to ensuring compatibility with renovisceral vessel morphology, a non-aneurysmal aortic segment of at least 5 mm in length between the distal edge of the SMA and the aneurysm is required to establish a minimum effective sealing zone of 20 mm.

**Table 1:** Proposed anatomic criteria for endovascular repair using the WeFlow-JAAA system.

Criteria	No.	%
*Patients (juxtarenal and pararenal)*	**170**	**100**
Infra-SMA neck < 5 mm	3	1.8
Aortic diameter at the celiac trunk origin < 18 mm or > 34 mm	3	1.8
Aortic diameter at the SMA origin < 18 mm or > 34 mm	2	1.2
Unhealthy proximal sealing zone	31	18.2
Extensive thrombus (> 5 mm thickness, > 50% of the aortic circumference)	22	12.9
Extensive calcium (> 50% of the aortic circumference)	1	0.6
Localized aneurysmal dilatation (> 34 mm)	4	2.4
Aortic ulcer or localized dissection within the sealing zone	4	2.4
Maximum tortuosity or angulation along the centerline of the proximal sealing zone and the tip of the bare stent segment (γ-angle) ≥ 60°	1^a^	0.6
Proximal sealing zone angulated ≥ 60° relative to the aortic segment harbouring the renal artery immediately below the proximal sealing zone (α-angle)	6^a^	3.5
Aortic diameter at the more cephalic renal artery < 18 mm	1	0.6
Aortic diameter at the bifurcation < 16 mm	2	1.2
Diameter of the SMA origin < 5 mm or > 12 mm	2	1.2
No bifurcated segment of the SMA < 10 mm	1	0.6
Diameter at the origin of any renal arteries < 4.5 mm or > 10 mm	47	27.6
Renal artery stenosis > 50%	20	11.8
Slender main trunk of the renal artery	1	0.6
No bifurcated segment of the renal artery < 10 mm	6	3.5
Accessory renal artery that supplies > 25% of each kidney or > 40% of one kidney	10	5.9
Take-off angle of the renal artery > 45° ^b^	1	0.6
Feasibility of the iliofemoral access (extreme tortuosity or bilateral stenosis < 7 mm)	7	4.1
*Ability to incorporate renovisceral arteries*	**125**	**73.5**
Longitudinal distance between the lower margin of the SMA and the upper margin of the renal artery ≥ 0 mm	153	90
Circumferential location incorporation of renal arteries	138	81.2
Circumferential location of the renal arteries with a superior margin < 5 mm below the lower margin of the SMA can range from 8–10 o’clock or 2–4 o’clock (60°-120° or 240°-300°)	39	22.9
Circumferential location of the renal arteries with a superior margin ≥ 5 mm below the lower margin of the SMA had no restrictions	99	58.2
Longitudinal distance between the lower margin of the celiac trunk and the upper margin of the SMA ≥ 5 mm	148	87.1
Circumferential location incorporation of celiac trunk	148	87.1
Configuration A: Celiac trunk arises from the aorta between 11:00 and 1:00 o’clock	145	85.3
Configuration B: Celiac trunk arises from aorta between 12:00 o’clock and 2:00 o’clock	155	91.2
*Suitability: conservative criteria*	**59**	**34.7**
*Suitability: liberal criteria*	**109**	**64.1**

^a^, one patient presented with both an α-angle and a γ-angle ≥ 60°, and the total number of unique patients with unsuitable angulation (either α-angle ≥ 60° or γ-angle ≥ 60°) was 6; ^b^, a positive take-off angle indicates that the renal artery origin is above the horizontal or transverse axis perpendicular to the aortic axis, corresponding to upward-oriented renal arteries.

**Fig. 3:**
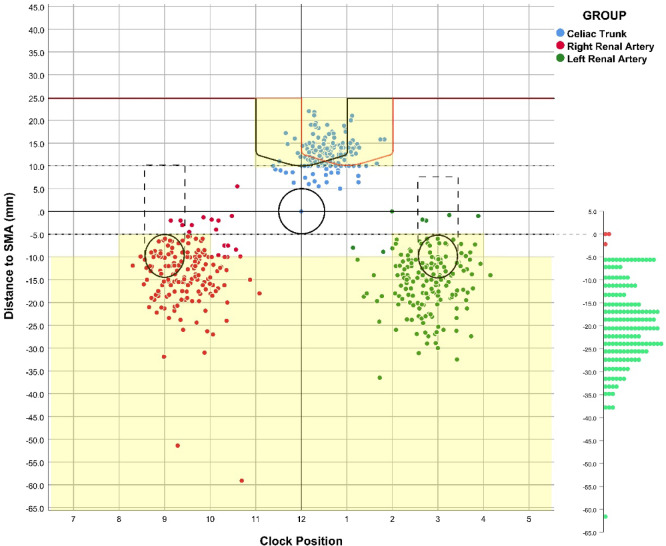
Positioning of the celiac axis (blue), right renal artery (red), and left renal artery (green) in relation to the SMA in configurations A and B of the proximal body of the WeFlow-JAAA system. The axial positions of the dots represent the longitudinal distances of the celiac trunk (lower margin) and renal arteries (upper margin) relative to the upper and lower margins of the SMA, respectively. The SMA take-off is positioned at 12 o’clock in all cases for feasibility evaluation. Overall, 73.5% of patients achieved successful renovisceral artery incorporation with both configurations of the WeFlow-JAAA system, as indicated by the yellow area. The dots on the right sidebar denote the most proximal extent of aneurysm among 170 patients with JRAAAs and PRAAAs. Red dots indicate aneurysms that are not suitable for repair within the device design’s anatomic recommendation, while green dots signify aneurysms within the anatomic guidelines. A proximal seal zone of ≥ 20 mm was achieved in 98.2% of patients (green dots).

The inner branch incorporates a pivot mechanism, defined by a small inner orifice (6–8 mm) coupled with a larger, offset outer orifice (Supplementary Figure S3). This non-planar design allows the branch to pivot, accommodating variability in renal artery take-off angles and axial misalignment, accommodating a wider range of renal artery take-off angles and suboptimal axial alignments compared to standard fenestrations (e.g., Cook Zenith Fenestrated) or external directional branches (e.g., Cook t-Branch). Although the flexibility of the pivot ostium of the inner branches theoretically mitigates the stringent clock position requirements for bilateral renal arteries, standardized criteria were implemented to ensure adequate sealing and to address potential operative challenges. The more cephalic renal artery, with a superior margin less than 5 mm below the lower margin of the SMA, was limited to 8–10 o’clock or 2–4 o’clock positions (as illustrated in Fig. 3). In contrast, the renal arteries with a superior margin at least 5 mm below the lower margin of the SMA were exempt from clock position restrictions. Renal arteries with a superior margin above the lower margin of the SMA were excluded from consideration.

For accessory renal arteries, exclusion was deemed appropriate if volumetric analysis via computed tomography indicated that the artery perfused less than 25% of each kidney or less than 40% of one kidney^9,15^.

Anatomic feasibility was assessed under two scenarios. The conservative instructions for use (IFU) criteria were satisfied if all proposed inclusion criteria were met and no exclusion criteria were present. The liberal IFU criteria were deemed satisfied when the experienced senior author, W.G., determined that an adequate seal could be achieved and all renovisceral arteries could be included. The liberal IFU criteria for the WeFlow-JAAA system specified a neck diameter of at least 18 mm and no more than 34 mm, a minimum distance of 5 mm between the upper margin of the aneurysm and the lower margin of the SMA, the ability to incorporate all renovisceral arteries, and the absence of non-sacrificable accessory renal arteries or early renal artery bifurcations (less than 10 mm). The aortic neck angle, thrombus burden of the proximal neck^16^, renal artery diameter, and difficult access were not considered essential criteria in the liberal IFU criteria, provided that an adequate seal could be achieved with the device.

### Statistical analysis

The primary endpoint of this study was the overall and category-specific anatomic feasibility based on the proposed conservative or liberal IFU criteria for patients with JRAAAs or PRAAAs. Continuous variables were presented as mean (SD) or median (range), as appropriate. Statistical analyses were conducted using SPSS software (version 26.0) and Microsoft Excel.

## Results

Between February 2022 and January 2024, a total of 170 patients were enrolled for imaging screening within the database of the GREAT study (NCT05179967), a multicenter clinical trial investigating the WeFlow-JAAA stent graft for JRAAAs and PRAAAs. All patients were included in our study, comprising 117 patients (68.8%) with JRAAAs and 53 patients (31.2%) with PRAAAs.

The anatomic characteristics of the aneurysms, as per the proposed criteria, are summarized in Table 1.

### Proximal sealing zone

Among the 170 patients with JRAAAs and PRAAAs, an effective proximal sealing zone of at least 20 mm was achieved in 98.2% (167/170) of patients (Fig. 3). An aortic neck diameter within the range of 18 to 34 mm was attained in 97.6% (166/170) of patients, while 3.5% (6/170) of patients were deemed unsuitable due to γ-angle ≥ 60°or α-angle ≥ 60°. An unhealthy proximal sealing zone was noted in 18.2% (31/170) of patients, including cases with extensive thrombosis (22/170), greater than 50% circumferential calcification (1/170), localized aneurysmal dilatation (4/170), and aortic ulcer or local dissection (4/170) within the coverage of the proximal sealing zone.

### Renovisceral artery incorporation

The locations of renovisceral arteries are depicted in Fig. 3. Configuration B successfully incorporated all renovisceral arteries in 70% (119/170) of patients, while configuration A achieved this in 64.7% (110/170) of patients. The overall rate of renovisceral artery incorporation was 73.5% (125/170). The longitudinal distance requirement from the upper margin of the SMA to the lower margin of the celiac trunk (≥ 5 mm) was met by 87.1% (148/170) of patients. Similarly, 81.2% (138/170) of patients fulfilled the criteria for both longitudinal distance and clock position of renal arteries relative to SMA. These two factors were the most significant considerations affecting the renovisceral artery incorporation using the WeFlow-JAAA system.

### Renal artery anatomy

22.4% (38/170) of patients had at least one accessory renal artery, with 5.9% (10/170) of patients displaying more than 25% perfusion of each kidney by accessory renal arteries. Early renal artery bifurcations (less than 10 mm) were observed in 3.5% (6/170) of patients. The renal artery diameter criteria (4.5–10 mm) were satisfied in 72.4% (123/170) of patients, which was the most common factor limiting the anatomic feasibility of the WeFlow-JAAA system according to the conservative IFU criteria.

### Other exclusion criteria

According to the conservative IFU criteria, 4.1% (7/170) of patients were deemed unsuitable for endovascular repair due to extreme tortuosity of the iliofemoral access or bilateral iliofemoral artery stenosis (less than 7 mm). An aortic diameter of at least 18 mm at the level of the more cephalic renal artery was absent in 0.6% (1/170) of patients, potentially leading to postoperative compression of the inner branches and subsequent occlusion of the renal artery. An aortic diameter of less than 16 mm at the aortic bifurcation was noted in 1.2% (2/170) of patients.

### Anatomic feasibility

The overall suitability of the WeFlow-JAAA system, according to the conservative and liberal IFU criteria, is summarized in Table 2. Anatomic feasibility was achieved based on conservative IFU criteria in 34.7% (59/170) of patients (95% CI: 27.5% to 41.9%). The most common anatomical exclusion criteria were inadequate renal artery diameter (27.6%, 47/170), failure to incorporate renal arteries (18.8%, 32/170), inability to incorporate the celiac trunk (12.9%, 22/170), and an unhealthy proximal sealing zone (18.2%, 31/170). Under liberal IFU criteria, 64.1% (109/170) of patients were deemed suitable (95% CI: 56.8% to 71.4%), with configuration A applicable in 56.5% (96/170) and configuration B applicable in 60.6% (103/170) of these suitable cases.

**Table 2:** Summary of strict and Liberal anatomic criteria for endovascular repair of juxtarenal and pararenal AAAs using the WeFlow-JAAA system.

Anatomic criteria	Conservative, %	Liberal, %
Ability to achieve a 20-mm proximal sealing zone	98.2	98.2
Renovisceral artery incorporation	73.5	73.5
Configuration A	64.7	64.7
Configuration B	70.0	70.0
Renal artery issues precluding endovascular repair	34.1	10
Absence of a healthy proximal sealing zone	18.2	2.4
Tortuosity of the proximal landing zone (γ-angle ≥ 60°)	0.6	0
Aortic neck angulation (α-angle ≥ 60°)	3.5	0
Others*	4.7	1.8
Suitability	34.7	64.1
Configuration A	32.9	56.5
Configuration B	32.4	60.6

*, “others” included the feasibility of the iliofemoral access, the diameter at the more cephalic renal artery, and the diameter at the aortic bifurcation.

A subgroup analysis was conducted to assess the anatomic feasibility between patients with JRAAAs (*n* = 117) and PRAAAs (*n* = 53). The suitability rate under conservative IFU was significantly higher for JRAAAs (38.5%, 45/117) compared to PRAAAs (26.4%, 14/53). A comparable trend was noted under liberal IFU criteria (70.1%, 82/117 vs. 50.9%, 27/53). The detailed results of this analysis are presented in Supplementary Table S1. An additional analysis stratified by maximum aortic aneurysm diameter demonstrated no significant impact on anatomic suitability, as visualized in Supplementary Fig. S4.

## Discussion

In this study, we evaluated the anatomic feasibility of the WeFlow-JAAA stent-graft system for treating patients with juxtarenal and pararenal AAAs. Our results indicated that, based solely on anatomic criteria, the WeFlow-JAAA system could offer endovascular repair to 64.1% of patients with JRAAAs or PRAAAs in clinical practice, provided there is a minimum of 5 mm of normal aorta below the SMA.

Our findings align with previous research concerning the anatomic suitability of OTS devices for JRAAAs and PRAAAs. Early preliminary studies reported an estimated anatomic feasibility of over 70% for the Cook p-Branch in patients who were preselected for customized devices, which may introduce selection bias^17^. In an unselected cohort of JRAAA and PRAAA patients, Mendes et al.^9^ found that, according to the conservative IFU criteria, the suitability of the Cook p-Branch and Endologix Ventana was 33% and 27%, respectively, which is consistent with our finding of 34.7%. However, when applying liberal IFU criteria, the overall anatomic suitability of the WeFlow-JAAA system appears comparable to the combined anatomical fit of the other two devices (64.1% vs. 63%^9^). This may be attributable to the superior proximal sealing capability of WeFlow-JAAA compared to the Ventana (98.2% vs. 88%^9^), as well as improved renovisceral artery incorporation relative to the p-Branch (73.5% vs. 61%^9^).

Ensuring adequate proximal seal and renovisceral artery incorporation is crucial for the effective clinical treatment of JRAAAs and PRAAAs using any endograft device. For fenestrated OTS devices, the major concern and anatomical limitation of longer proximal sealing zone designs, such as the p-Branch, is the incorporation of renal axial or circumferential position^18,19^. In contrast, a significant exclusion criterion for simpler designs with two fenestrations, like the Ventana, is the restriction on infra-SMA neck length^19^. Long-term outcomes of customized fenestration devices have raised concerns about relying solely on renal artery fenestrations to provide a durable seal beyond 5 to 10 years^20^. In comparison, the design of WeFlow-JAAA, which combines fenestrations with inner branches, may mitigate the limitations associated with simple fenestration designs for JRAAAs and PRAAAs. The inclusion of inner branches with pivot mechanisms can minimize restrictions on renal axial or circumferential positioning, which may explain the superior renovisceral artery incorporation observed with WeFlow-JAAA system in our study. Furthermore, the inner branches with pivot design can significantly reduce the anatomical limitations related to early bifurcation and take-off angle of renal arteries compared to fenestration designs. However, it is crucial to emphasize that these findings are derived from a cohort within the Chinese population. Given that anatomic characteristics may exhibit ethnic variations, the generalizability of our results to other populations necessitates further validation in international cohorts.

When assessing the anatomical suitability of the WeFlow-JAAA system for clinical application, it is essential to balance between feasibility and effectiveness. In this study, we established both conservative and liberal IFU criteria to evaluate the real-world clinical applicability of this novel OTS device. In practice, we anticipate that the clinical applicability of the WeFlow-JAAA, which incorporates a pivot design along with the sealing effects from the inner branches of both renal arteries, will exceed the outcomes reported in our study. However, it is important not to overextend the anatomical feasibility, as this may lead to unpredictable complications and risks. For example, although some have suggested expanding the suitability by intentionally covering the celiac trunk, this approach carries the potential risk of immediate or delayed visceral ischemia and type II endoleak (persistent retrograde celiac artery flow)^21–23^. Additionally, including cases where the superior margin of the renal artery exceeds the inferior margin of the SMA to broaden the feasibility may pose challenges in inner-branch catheterization and implantation. This could promote upward migration of the system and subsequently create a sharper angle between the SMA fenestration and the aorta, thereby increasing the risk of type IIIc endoleak^15^. In our study, the subgroup analysis revealed significantly higher anatomic suitability for JRAAAs compared to PRAAAs. The increased prevalence of complex renal artery anatomy and unhealthy proximal sealing zone in PRAAAs likely contributed to this disparity. Therefore, to ensure patient safety and procedural success, careful consideration must be given to the potential complications associated with expanding the anatomic criteria before proceeding with such cases.

In China, endovascular repair of JRAAAs and PRAAAs currently relies heavily on physician-modified endografts, which are unsuitable for emergency cases due to the time required for manual modification and carry potential legal and long-term durability concerns. The WeFlow-JAAA system, as the first investigational off-the-shelf device in China specifically designed for JRAAAs and PRAAAs, has the potential to significantly influence clinical decision-making. Its immediate availability may present a timely endovascular option for managing ruptured or impending-ruptured complex AAAs, potentially enhancing outcomes in emergency settings while offering a standardized and regulated alternative to physician-modified solutions.

This study has several limitations. Firstly, the sample size was not sufficiently large, which may affect the generalizability of our findings. Secondly, as our study cohort was derived from a screening population for a clinical trial, it may not fully represent the broader population of patients with JRAAAs and PRAAAs, particularly those with a short healthy neck below the SMA (less than 5 mm), who might be more suitable for a multibranched OTS device such as G-Branch^24^. However, it is important to note that this study cohort provides valuable insights into real-world clinical practice, as it consists of individuals with genuine clinical treatment needs encountered in a national multicenter clinical trial investigating WeFlow-JAAA without strict selection criteria. Furthermore, the enrollment of this screening population was conducted in a blinded manner during the imaging analysis, helping to minimize potential measurement bias.

## Conclusions

The WeFlow-JAAA endograft system exhibits a suitability rate of 64.1% for treating JRAAAs and PRAAAs with a healthy sealing zone of at least 5 mm below the SMA within the Chinese population. Nevertheless, a persistent limitation remains in achieving optimal incorporation of the renovisceral artery. Future research should focus on enhancing the system’s adaptability to accommodate more complex aortic anatomical variations, thereby improving patient outcomes and broadening the clinical application of this OTS device.

## Supplementary Information

Below is the link to the electronic supplementary material.

Supplementary Material 1

## Data Availability

The data that support the findings of this study are available from the corresponding author upon reasonable request. Due to privacy concerns, the data is not publicly available. Interested researchers are encouraged to contact the corresponding author for further information regarding data access.
