# Supplementary material for: Anatomic feasibility of the WeFlow-JAAA endograft system for treating juxtarenal and pararenal abdominal aortic aneurysms
Source: Sci Rep. 2025 Nov 13;15:39818. doi: 10.1038/s41598-025-23485-y (PMC12615794; doi:10.1038/s41598-025-23485-y)
Supplement: Supplementary file 1 — Supplementary Material 1 [file 41598_2025_23485_MOESM1_ESM.docx]

Supplementary Materials for

**Anatomic Feasibility of the WeFlow-JAAA Endograft System for Treating Juxtarenal and Pararenal Abdominal Aortic Aneurysms**

Jiang-Ping GAO, Chun-An-Sheng WANG, Hong-Peng ZHANG, Li-Jun WANG, Wei GUO*

**This PDF file includes:**

**Figure S1:** Flow diagram of cases included in this study.

**Figure S2:** Illustrations of γ-angle and α-angle.

**Figure S3:** The pivot mechanism of the renal inner branch (outside view and lateral view)

**Figure S4:** Anatomic suitability of the WeFlow-JAAA stratified by maximum aortic aneurysm diameter.

**Table S1:** Summary of strict and liberal anatomic criteria for endovascular repair of juxtarenal and pararenal AAAs using the WeFlow-JAAA system


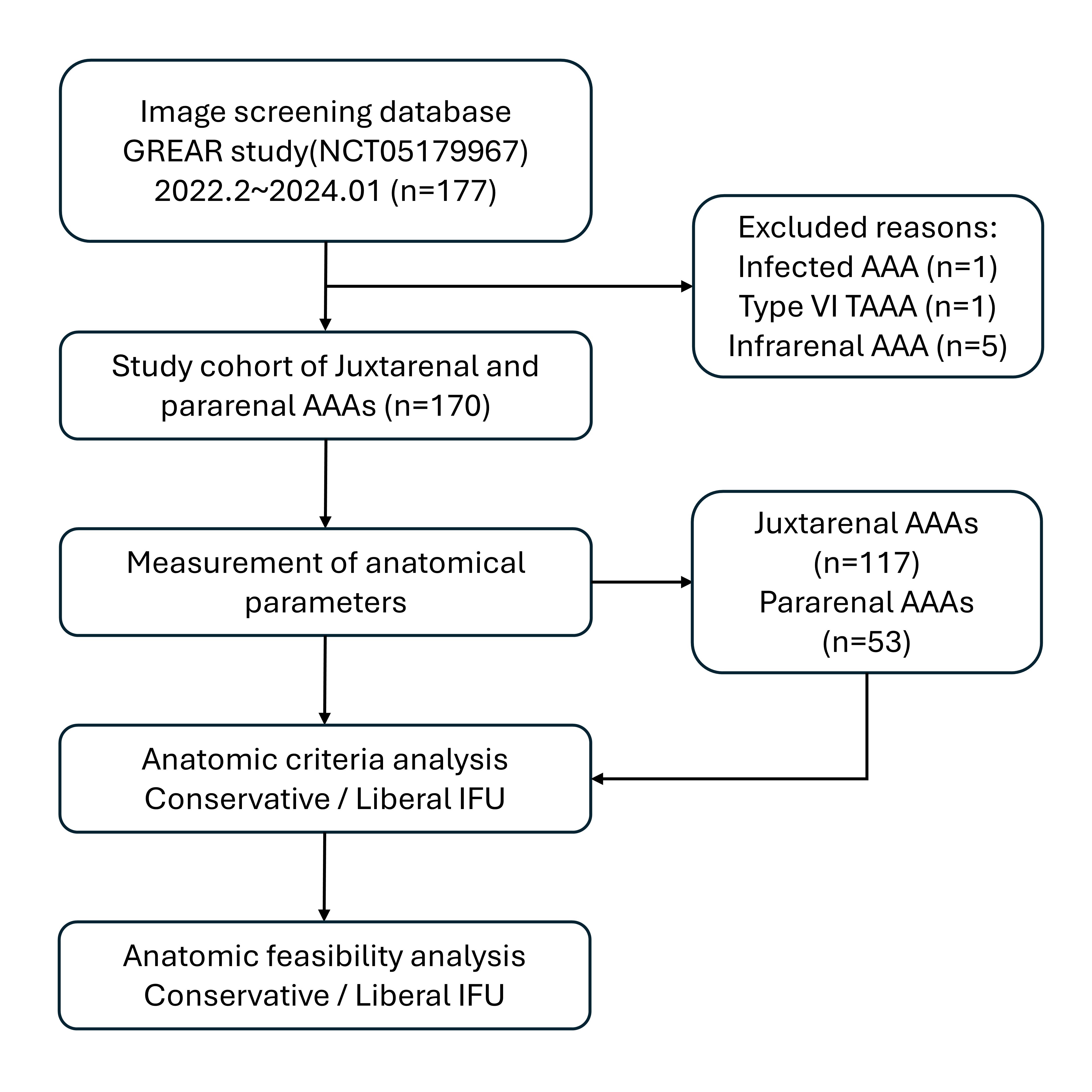


**Figure S1.** Flow diagram of cases included in this study. AAA, abdominal aortic aneurysm; IFU, instruction for use; TAAA, thoracic abdominal aortic aneurysm.


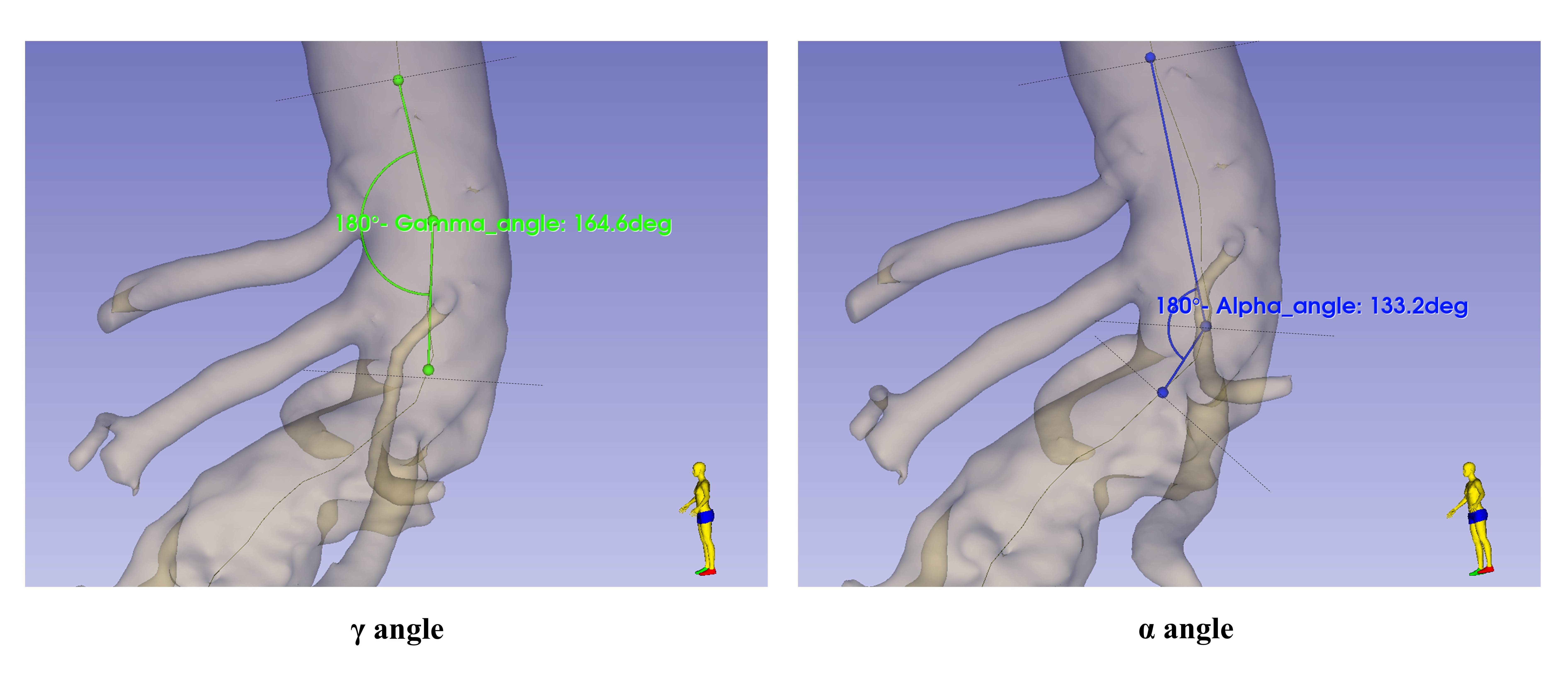


**Figure S2**. Illustrations of γ-angle and α-angle. The γ-angle was defined as the maximum tortuosity or angulation along the centerline of the proximal landing zone, spanning from 33-35 mm proximally to the superior margin of the SMA to 5 mm below the lower margin of the SMA. This measurement encompassed both the proximal sealing zone and the bare stent segment, playing a crucial role in ensuring optimal stent adherence to the aortic wall following the procedure. The α-angle was defined as the maximum angle between the proximal sealing zone and the aortic segment harbouring the renal artery immediately below the proximal sealing zone, which is significant for the successful implantation as well as the long-term patency of the renal artery branches.


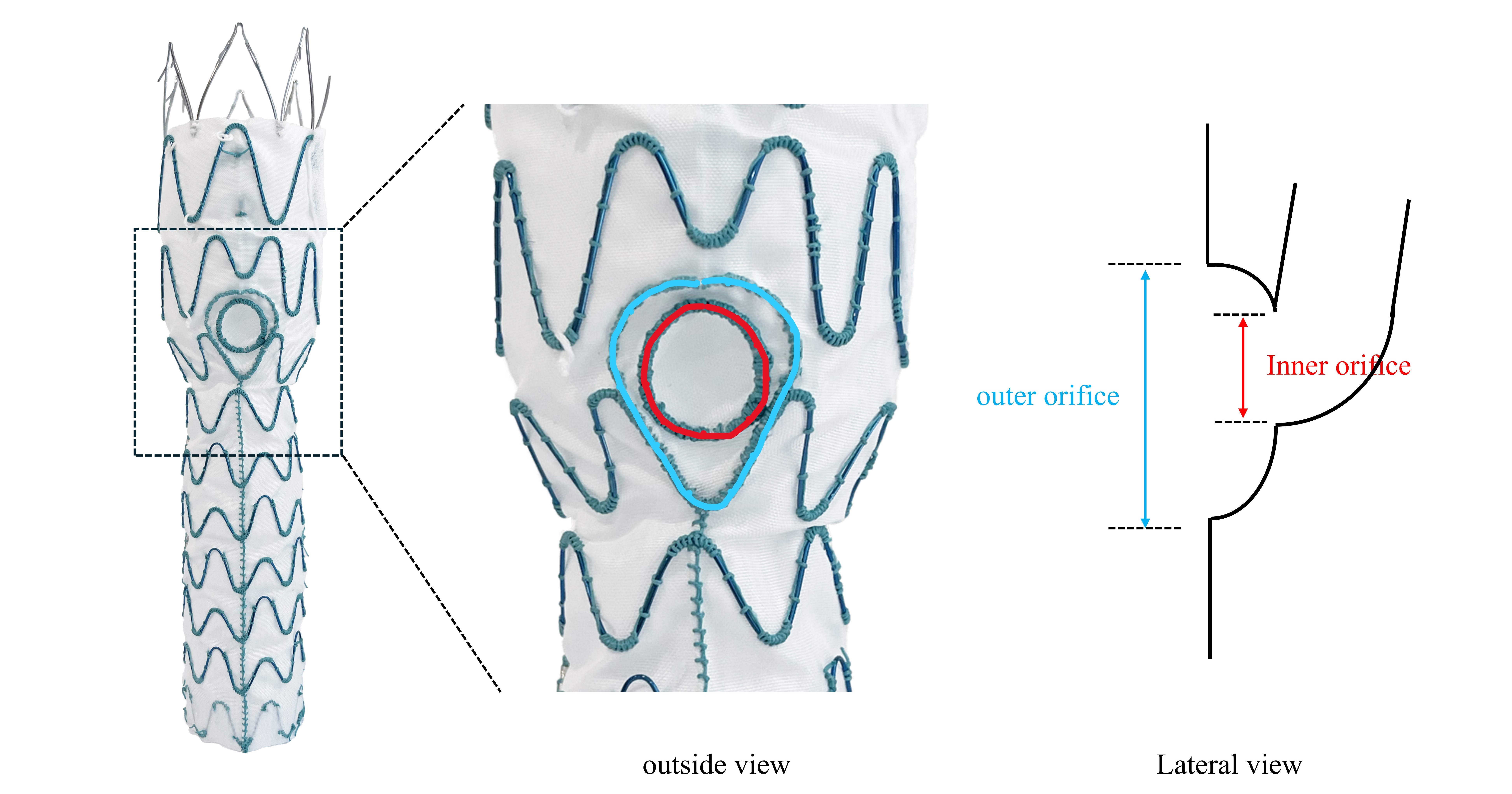


**Figure S3.** The pivot mechanism of the renal inner branch (outside view and lateral view) was defined by a small inner orifice (6-8mm) coupled with a larger, offset outer orifice. This non-planar design allows the branch to pivot, accommodating variability in renal artery take-off angles and axial misalignment, accommodating a wider range of renal artery take-off angles and suboptimal axial alignments compared to standard fenestrations (e.g., Cook Zenith Fenestrated) or external directional branches (e.g., Cook t-Branch).


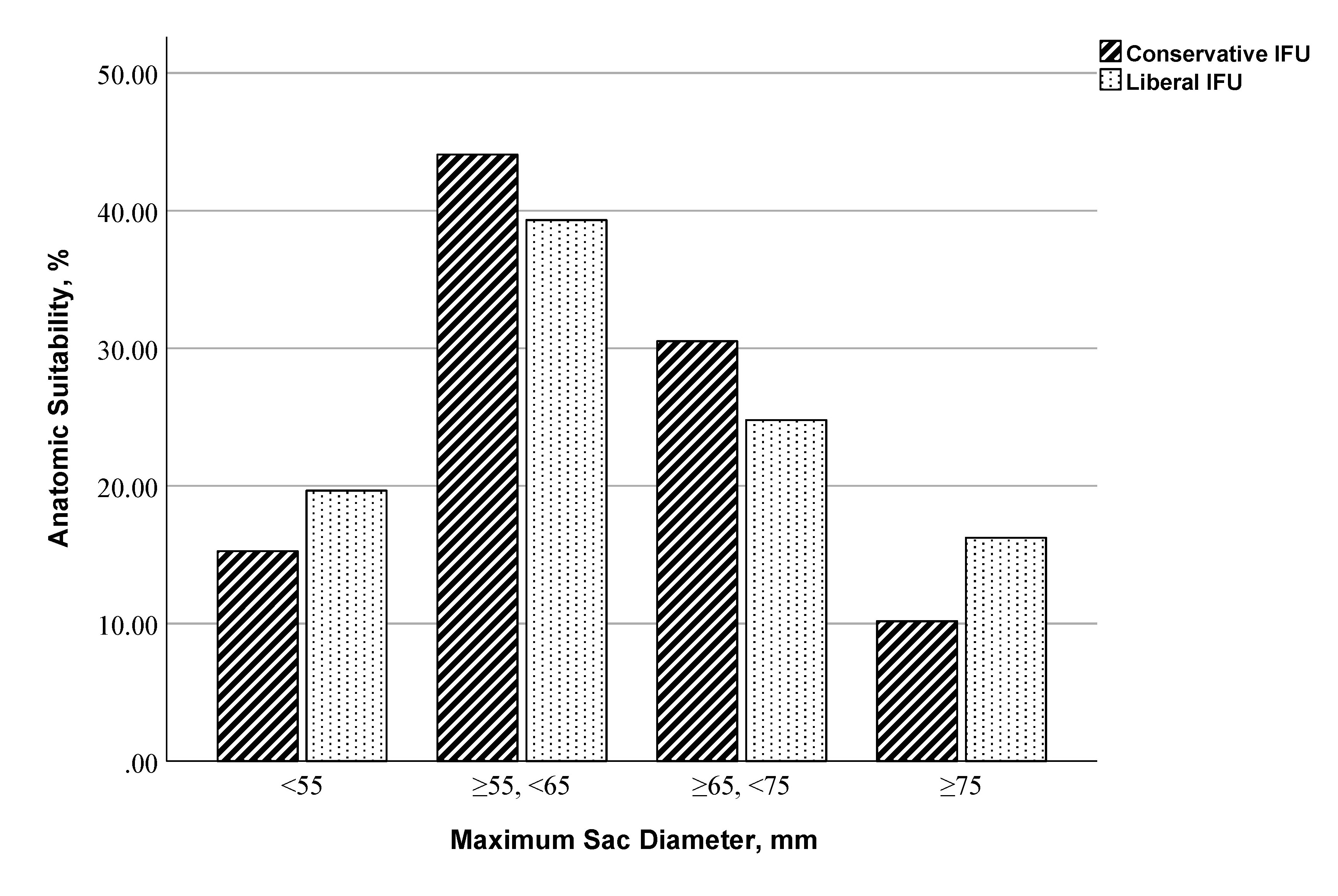


**Figure S4**. Anatomic suitability of the WeFlow-JAAA stratified by maximum aortic aneurysm diameter. Patients were categorized into four groups based on maximum aneurysm diameter: <55 mm (n=34), ≥55, <65 mm (n=67), ≥65, <75 mm (n=39), and ≥75 mm (n=30). It seems that no significant association was observed between aneurysm diameter and anatomic feasibility. The distribution of patients across groups approximated a normal distribution, with most cases concentrated in the ≥55, <65 mm and ≥65, <75 mm ranges.

**Table S1.** Summary of strict and liberal anatomic criteria for endovascular repair of juxtarenal and pararenal AAAs using the WeFlow-JAAA system.

|  | *Juxtarenal AAAs* | | | *Pararenal AAAs* | |
| --- | --- | --- | --- | --- | --- |
| *Anatomic criteria* | *Conservative* | *Liberal* | *Conservative* | | *Liberal* |
| Ability to achieve a 20-mm proximal sealing zone | 100.0% (117/117) | 100.0% (117/117) | 94.3% (50/53) | | 94.3% (50/53) |
| Renovisceral artery incorporation | 77.8% (91/117) | 77.8% (91/117) | 64.2% (34/53) | | 64.2% (34/53) |
| Configuration A | 69.2% (81/117) | 69.2% (81/117) | 54.7% (29/53) | | 54.7% (29/53) |
| Configuration B | 73.5% (86/117) | 73.5% (86/117) | 62.3% (33/53) | | 62.3% (33/53) |
| Renal artery issues precluding endovascular repair | 30.8% (36/117) | 7.7% (9/117) | 41.5% (22/53) | | 15.1% (8/53) |
| Absence of a healthy proximal sealing zone | 14.5% (17/117) | 1.7% (2/117) | 26.4% (14/53) | | 3.8% (2/53) |
| Tortuosity of the proximal landing zone (γ-angle ≥60°) | 0.9% (1/117) | 0 | 0 | | 0 |
| Aortic neck angulation (α-angle ≥60°) | 1.7% (2/117) | 0 | 7.5% (4/53) | | 0 |
| Others* | 5.1% (6/117) | 0.9% (1/117) | 3.8% (2/53) | | 3.8% (2/53) |
| Suitability | 38.5% (45/117) | 70.1% (82/117) | 26.4% (14/53) | | 50.9% (27/53) |
| Configuration A | 36.8% (43/117) | 63.2% (74/117) | 24.5% (13/53) | | 41.5% (22/53) |
| Configuration B | 35.9% (42/117) | 65.8% (77/117) | 24.5% (13/53) | | 49.1% (26/53) |

*, “others” included the feasibility of the iliofemoral access, the diameter at the more cephalic renal artery, and the diameter at the aortic bifurcation.
